# Audio-Guided Mindfulness Meditation During Transcranial Magnetic Stimulation Sessions for the Treatment of Major Depressive Disorder: A Pilot Feasibility Study

**DOI:** 10.3389/fpsyg.2021.678911

**Published:** 2021-08-17

**Authors:** Fiamma Cavallero, Michael C. Gold, Eric Tirrell, Fatih Kokdere, Nancy Donachie, Dan Steinfink, Joseph Kriske, Linda L. Carpenter

**Affiliations:** ^1^Butler Hospital TMS Clinic and Neuromodulation Research Facility, Providence, RI, United States; ^2^Brown Department of Psychiatry and Human Behavior, Warren Alpert Medical School at Brown University, Providence, RI, United States; ^3^Salience TMS Neuro Solutions, Plano, TX, United States

**Keywords:** depression, TMS, mindfulness, meditation, MBCT, MDD

## Abstract

**Background:** Mindfulness-Based Cognitive Therapy (MBCT) has been shown to enhance the long-term treatment outcomes for major depressive disorder (MDD), and engagement of specific brain activities during brain stimulation may produce synergistic effects. Audio-guided meditation exercises are a component of MBCT that might be combined with standard transcranial magnetic stimulation (TMS) therapy sessions. We developed and pilot-tested a modified MBCT protocol for patients undergoing a standard course of TMS for MDD.

**Methods:** Four MBCT audiotracks with differing durations and types of mental focus were selected. Patients listened to the audiotapes through headphones during daily TMS sessions for 5 consecutive weeks. The primary goal was to evaluate the feasibility and acceptability of the meditation intervention with TMS. Changes in self-rated measures of symptom severity, stress, life satisfaction, and mindfulness were also assessed.

**Results:** Seventeen depressed subjects completed the study and 12 terminated early. Reasons for discontinuation included an inability to meditate in the treatment setting and induction of negative mood states. TMS percussive sensations and clicking sounds hindered the ability of patients to fully concentrate on or hear the voice of the audiotape narrator. Some became overwhelmed or felt increased pressure, anxiety, or aggravation trying to do meditation exercises while receiving TMS.

**Conclusion:** There is a growing interest in combining TMS with other concurrent psychotherapeutic interventions to optimize treatment outcomes. The results highlight numerous feasibility issues with MBCT *via* guided audiotapes during TMS treatment. Future work should draw on these shortcomings to evaluate the appropriateness of MBCT for depressed patients undergoing neuromodulation.

## Introduction

Major depressive disorder (MDD) has a 16% lifetime prevalence (Kessler and Bromet, 2013) among the world population. The WHO revealed that it is a leading cause of disability (Moussavi et al., 2007). The risk of relapse following a single depressive episode is 50%, and then the risk drastically escalates to 90% after the third episode (Kessing, 2003). Depressed patients face a significant, chronic psychosocial impairment, and treatment-resistant populations face a higher risk of suicide (Hawton et al., 2013).

There is still a need for interventions to address a growing public health burden associated with chronic, recurrent, and pharmacoresistant forms of depressive illness. Transcranial magnetic stimulation (TMS) therapy is a noninvasive brain stimulation method that involves the delivery of magnetic pulses through the skull to stimulate proximal cortical neurons (Barker et al., 1985), which in turn activate more distal brain regions in specific functional networks (Philip et al., 2018). Repetitive TMS (rTMS) therapy is an FDA-approved intervention for MDD based on sham-controlled clinical trials where the stimulation of the left dorsolateral prefrontal cortex (L-DLPFC) over 4–6 weeks demonstrated efficacy in patients whose symptoms were not relieved by traditional antidepressant medications (George et al., 2000; O'Reardon et al., 2007). In the United States, TMS therapy is most commonly provided as a course of five 30-min sessions/week for 6 weeks, followed by a taper phase of six treatments over an additional 3 weeks. Patients have been shown to maintain the antidepressant response to TMS therapy 6 months poststimulation (Concerto et al., 2015).

While TMS alone is an effective therapy for depression, recent work has investigated the effect of TMS and various concurrent (i.e., during stimulation) tasks to improve clinical outcomes. Psychotherapy performed during the 30 min, daily TMS sessions appeared to show greater depression improvement than TMS alone in a preliminary study (Donse et al., 2018), and symptom provocation during TMS sessions was superior to TMS alone for treating obsessive-compulsive disorder (Isserles et al., 2011). Preliminary success was also reported for combining exposure therapy with simultaneous TMS for post-traumatic stress disorder (PTSD) (Fryml et al., 2019). These approaches build on a large body of nonclinical literature displaying the cognitive enhancements induced by online, high-frequency stimulation during learning and tasks (reviewed by Luber and Lisanby, 2014). Cognitive enhancements and depression therapeutic mechanisms of TMS are believed, at least in part, to be related to network circuit modulation (Eshel et al., 2020), which may display therapeutic synergy when combined with functional tasks by capitalizing on state-dependent effects of stimulation (Sathappan et al., 2019). Additionally, induction of neuroplasticity and metaplasticity of TMS appears related to MDD pathology (Cantone et al., 2017). This pathology, particularly cognitive impairment in depression, has been linked to hemodynamic changes in patients with late-life depression (Puglisi et al., 2018).

Beyond psychotherapy, mindfulness-based interventions for psychiatric disorders have grown in popularity over the last decade. Mindfulness is characterized as a mental state that incorporates intentionality, focus on the present moment, bodily awareness, and acceptance (Grossman, 2015). Prominent within this framework is Mindfulness-Based Cognitive Therapy (MBCT), a revision to Cognitive-Based Therapy (CBT) for patients with MDD.

Mindfulness-Based Cognitive Therapy extrapolates the foundations of CBT to modulate the relationship between depressed states and their underlying dysfunctional bias and the ruminative thought patterns (Segal et al., 2013). MBCT is based on formal and informal mindfulness practices, such as body scans, sitting meditation, breathing, focused stretching and movement, mindful walking, and guided exercises in working with difficulty. MBCT is specifically tailored for individuals with depression and as an intervention to prevent recurrent depressive episodes. During daily, independently practiced 20-min sessions, MBCT exposes subjects to the concepts of compassionate awareness, mindful acceptance, skillful response, and moment-to-moment focus. These practices are hypothesized to re-orient negative patterns of thinking associated with MDD symptom burden (Segal et al., 2013).

The efficacy of MBCT protocols for preventing relapse in treatment-resistant depression was demonstrated in several long-term studies that compared it with treatment as usual (TAU) (Teasdale et al., 2000; Kuyken et al., 2008; Segal et al., 2010). However, in a study of patients who had experienced three or more depressive episodes, MBCT only displayed a superior benefit for those with a history of childhood trauma (Williams et al., 2014). The National Institute of Health and Clinical Excellence of United Kingdom recommends MBCT for patients who have experienced more than two major depressive episodes (Pilling et al., 2009). Despite the positive reports and endorsements, there has been a call for improved documentation of potential adverse effects of mindfulness practices in neuropsychiatric patient populations (Van Dam et al., 2018). Furthermore, it remains unseen how comorbid psychiatric conditions may impact the outcomes of MBCT in patients (Goyal et al., 2014).

Despite the growing interest in the combination of mindfulness-based training with low-intensity electrical brain stimulation (Badran et al., 2017; Monnart et al., 2019), none to date has systematically explored the feasibility of rTMS therapy with MBCT. Previous study has successfully administered TMS therapy with other concurrent psychotherapy interventions (e.g., Donse et al., 2018; Kozel et al., 2018; Russo et al., 2018), with the goals of optimizing clinic time for patients seated for their daily TMS treatments and capitalizing on the potential synergy of concurrent brain stimulation during the engagement of specific neural networks (Sathappan et al., 2019).

In this study, we conducted a dual-site feasibility study of audiotrack-guided MBCT performed during daily TMS for depression. Given the increasing interest in MBCT, the prior feasibility of combined rTMS with concurrent psychotherapies, and the routine ability of patients to listen to music or audio files during standard TMS, we hypothesized that MBCT during stimulation may be feasible. We developed self-rating scales to assess the usefulness of MBCT for patients with TMS and implemented standardized mindfulness self-report questionnaires to measure changes in mindfulness throughout the MBCT/TMS treatment duration. To represent the range of environments and patient samples where rTMS/MBCT might eventually be used, we evaluated the feasibility in two different TMS treatment settings (one academic hospital outpatient clinic and one private community clinic), and we did not place any constraints on the specific stimulation protocol the psychiatrist prescribed for depressed patients receiving treatment in their clinic. The goal was to assess the feasibility of the combined approach in such a way that would inform a future clinical trial if the intervention proved feasible and promising; the current work was neither designed nor powered to assess the possible therapeutic synergy of the concurrent TMS and MBCT.

## Methods

### Study Design and Objective

We conducted an open-label, single-arm pilot study to evaluate the feasibility and preliminary effects of combining a modified MBCT with standard rTMS in two naturalistic treatment settings (Butler Hospital TMS Clinic in Providence, RI, and Salience TMS Neuro Solutions in Plano, TX). Several MBCT elements were modified for adaptation to TMS treatment settings and to make the intervention suitable for individuals currently experiencing severe depression. The proportion of the sample completing the protocol and specific feedback provided by participants on a survey administered at the endpoint (or study termination) were the primary outcomes of interest. The study was approved by the Butler Hospital Institutional Review Board (IRB), and consent was obtained on IRB-approved consent forms.

### Study Participants

Study participants (*n* = 27) were recruited from consecutively treated patients who were starting a course of TMS therapy for MDD in the specialty clinics of Butler Hospital (*n* = 16) or Salience TMS Neuro Solutions (*n* = 11). The TMS patient population consisted of depressed individuals referred by themselves or by their clinicians. All had a primary diagnosis of nonpsychotic MDD and a history of nonresponse following one or more standard psychopharmacological medication trials in the current depressive episode. It was recommended that patients be maintained on their psychotropic medication regimen throughout their courses of TMS. Many of these patients also continued in ongoing psychotherapy with their community clinicians during their course of TMS and participation in this study.

Eligible patients were 18 years or older and able to understand the English language. Patients with TMS were invited to participate in this study by a research staff member during their 1st week of treatments. Enrolled participants were visually monitored for adverse events during their combined MBCT+TMS sessions and to ensure that they had adequate control over the audio tracks played on a portable electronic device (e.g., MP3 player, iPod, and computer tablet). They were encouraged to notify staff of any distress related to study procedures and reminded that they could refuse to answer any questions and/or decline to listen to meditation recordings on any day during their course of TMS therapy.

### Procedures

#### TMS Therapy

Stimulation protocol was chosen by the prescribing psychiatrists at each site and reflected the standard clinical practice of each clinic. At Butler Hospital, all patients were treated on a Neurostar device with a figure-8 coil (Neuronetics, Malvern, PA, United States). Treatment sessions were initiated with the standard “on-label” 10-Hz protocol over the L-DLPFC at 120% intensity relative to resting motor threshold (MT), for a minimum of 3,000 pulses. MT was determined by sending single pulses targeted at the left motor cortex to obtain the minimum intensity necessary to elicit 5/10 visible twitches in a finger of the contralateral hand. L-DLPFC was localized as the site 5.5 cm anterior to the MT location. The MT was established at the beginning of a treatment course and rechecked as needed to address tolerability or if there were changes to medications. Switch to an alternative (5-Hz) protocol (still targeting l-DLPFC) occurred in situations with poor tolerability assessed with the patient by TMS clinical staff (i.e., application site discomfort, residual headache) that did not subside within several days, or in cases of TMS-emergent activation (anxiety, insomnia, restlessness, and irritability) (Philip et al., 2016).

At the Salience site, all patients were treated on a MagPro device with a figure-8 coil (MagVenture, Farum, Denmark). Each treatment session was delivered as 1-Hz stimulation delivered to the right DLPFC at 120% intensity for 360 total pulses, followed by 20-Hz stimulation delivered to the L-DLPFC at 120% intensity for an additional 1,200 total pulses (Cash et al., 2017; Stubbeman et al., 2018). MT determination, DLPFC localization, and MT monitoring procedures were all identical to those at Butler Hospital.

#### Protocol of MBCT Modified for Combination With TMS

Participants listened to audio-guided meditation exercises while their TMS treatments were being delivered. Audio recordings provided upon purchase of the original MBCT manual (Segal et al., 2013) were downloaded onto electronic devices. To accommodate participants who preferred or were not able to complete guided meditation exercises exceeding 20 min for the final 3 weeks, we also used shorter versions of the same exercises downloaded from a website that provides free MCBT resources (freemindfulness.org).

Introduction to the study occurred during the 1st week of TMS treatments. To allow each patient with TMS an opportunity to accommodate the clinical routine and the uncomfortable sensation of magnetic pulses on their head, MBCT was not administered during the 1st week of TMS therapy. The original 8-week MBCT protocol was adjusted so the participant would listen to the audio recordings during daily sessions for only 5 weeks.

As a proxy for the weekly patient-clinician encounter described in the original MBCT protocol, the research staff provided additional guidance and explanations about the goals of MBCT and discussed any concerns arising with the practice of MBCT, before and after every treatment session. Self-administered audio-guided MBCT began after TMS session #5 as brief (5 min) meditation exercises, followed by an increase in duration gradually overtime for the remaining weeks, such that meditations during the final week of study participation were nearly as long as the treatment sessions themselves (i.e., 37 min at Butler, 20 min at Salience). Audiotapes were of a male voice (with background music or tones) speaking slowly, providing narration and instruction to guide the focus and attentional processes of the subject. Audio volume was user-dependent and adjusted by participants until they could hear the content of the meditation tracks through provided headphones. During the first few weeks, participants were encouraged to continue to meditate for the remainder of time left in their TMS treatment session after the recording ended.

The 5-week protocol of combined TMS and mindfulness training included the following:

*Week 1*: Audio-guided breathing exercise (5 min/day for 2 days) recognizing the breath as an anchor point to develop awareness, avoid judgment and rumination; audio-guided sitting meditation (10 min/day for 3 days) to explore thoughts, physical sensations, and feelings.

*Week 2*: Audio-guided sitting meditation (20 min/day) as an extended version of the previous sitting meditation. Consistent with the original MBCT protocol and associated with positive long-term results, research staff suggested that participants introduce a 20-min practice into their daily routines (outside of the TMS treatment setting).

*Week 3:* Audio-guided meditation on working with difficulty (25 min/day) to observe how negative patterns of thought become consistent, gain perspective, and decenter from both negative ideas and emotions.

*Week 4:* Audio-guided body scan (39 min/day at Butler; 20 min/day at Salience) familiarizing the subject with bodily discomfort and physical sensations by engaging and disengaging attention to prevent mental wandering.

*Week 5:* Audio-guided sitting meditation (36 min/day at Butler; 20 min/day at Salience) in its longer version, as an example of standard formal practice.

### Measures

Depression symptom severity was routinely assessed at baseline and serially after every five sessions during TMS treatment as standard practice in both clinics. Measures included the Inventory of Depressive Symptomatology (IDS-SR) (Rush et al., 2000) and the Patient Health Questionnaire (PHQ-9) (Kroenke and Spitzer, 2002).

Feasibility and mindfulness measures were administered at the start of participation in the study and again after the final TMS session, or earlier when a participant elected to terminate participation in study MBCT procedures prior to finishing their course of TMS therapy. Measures included the Perceived Stress Scale (PSS), a 14-item scale inquiring how stressful things have been in the past week (Cohen et al., 1983); the Multidimensional Assessment of Interoceptive Awareness (MAIA), a 37-item scale assessing body awareness with eight subscales (Mehling et al., 2018); the Quality of Life Enjoyment and Satisfaction Questionnaire-Short Form (Q-LES-Q-SF), a 16-item scale investigating the degree of enjoyment and satisfaction in daily functioning (Endicott et al., 2016); and the Five Facet Mindfulness Questionnaire (FFMQ), a 39-item measure consisting of five subscales about mindfulness core aspects (observing, describing, acting with awareness, non-judging and non-reactivity to inner experience) (de Bruin et al., 2012).

We developed a simple self-report questionnaire to gather data about how much the participant engaged in meditation practice outside of the TMS clinic during participation in the study. This self-report questionnaire was completed three times during the trial, at the end of weeks 3, 4, and 5. A feasibility questionnaire at the time of study exit assessed the strengths and weaknesses of the modified MBCT treatment intervention during TMS. This measure contained both closed and open-ended questions that captured the previous level of meditation experience of the participant, the level of ease or difficulty they had engaging in MBCT+TMS sessions (with the identification of specific obstacles or challenges the participant faced), their degree of success in continuing meditation during TMS after the narrated recording ended, their perceived degree of ease in meditation with increasing experience during the weeks in the study, the degree of subjective benefit derived from participation, the desirability of having combined MBCT+TMS during future courses of treatment, and whether they anticipated they would continue practicing meditation after study completion/termination.

### Statistical Analyses

Simple descriptive statistics were used to characterize the clinical and demographic features and responses of participants on the feasibility questionnaire. Paired *t*-tests were used to evaluate the baseline to endpoint scores on depression severity and stress/mindfulness measures. Possible differences between sites were evaluated with *t-*tests and chi-square test, as appropriate. Study completers were similarly compared with study dropouts on baseline features. Alpha was set to 0.05 for statistical significance, and tests were two-tailed. *P* values were Bonferroni-corrected for multiple comparisons, where appropriate.

## Results

**Figure 1:**
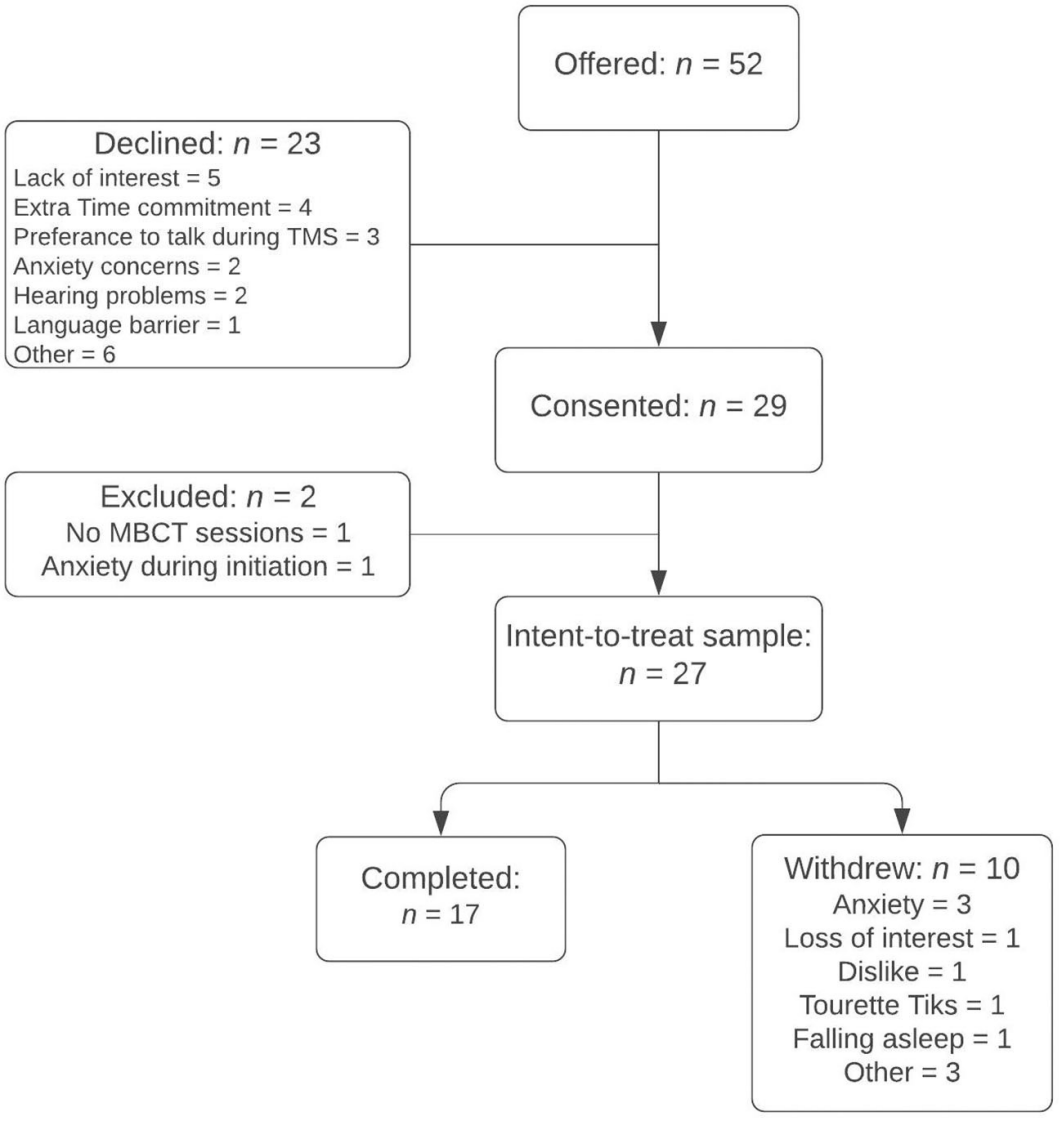
Participant Flowchart. MBCT = Mindfulness-Based Cognitive Therapy.

Participation in this pilot study was offered to 52 patients with MDD starting a course of TMS therapy across the Butler and Salience sites (Figure 1). Of the 23 (44%) of subjects who declined to participate, reasons cited were lack of interest (*n* = 5), extra time commitment (*n* = 4), preference to talk during TMS sessions (*n* = 3), concerns for heightened anxiety level with meditation (*n* = 2), hearing problems (*n* = 2), previous ineffective meditation trials (*n* = 2), language barrier (*n* = 1), and other diagnostic or treatment intolerability issues (*n* = 3). Of 29 patients who consented, two did not engage in at least one audio-guided meditation due to poor auditory acuity (*n* = 1) and excessive anxiety associated with the initiation and sensation of TMS treatments (*n* = 1); an “intent-to-treat” sample of 27 patients (*n* = 16 at Butler and *n* = 11 at Salience) was, thus, defined by those who practiced at least one session of audio-guided MBCT during TMS (Figure 1). Of these, 24 completed the feasibility questionnaire upon the study exit. Three patients, all of whom withdrew from the study prior to completion, did not complete the feasibility questionnaire.

As shown in Table 1 across both sites, 19 (70.4%) were female, with mean ± SD age of 48.4 ± 15.2 years. The group had moderate to severe depressive symptom severity at baseline, as reflected by mean ± SD scores of 43.11 ± 11.6 on IDS-SR and 17.2 ± 5.8 on PHQ-9. A total of 13 (48.1%) had a history of previous psychiatric hospitalization(s) and three (11.1%) had received at least one previous electroconvulsive therapy (ECT) session during the current or a past depressive episode. Participation in this study occurred during their first course of TMS for 18 (66.6%) subjects. Data from the 24 who completed the questionnaire at the time of study termination revealed that the vast majority (17; 71%) had previously practiced meditation, with a mean self-rating of 3.5 ± 2.9 for the level of prior experience with meditation, on a scale from 0 (no experience) to 10 (very experienced). Participants completed an average of 35 ± 7.7 TMS treatment sessions during their course of therapy, which included 18 ± 8.6 sessions of TMS+MBCT. Among these study demographics and treatment features, only baseline IDS-SR depression severity significantly differed between study sites (Butler Hospital: 47 ± 11; Salience: 37 ± 10. *p* = 0.03, *t* = 2.29); patients at the Butler site were more symptomatic at the start of their course of TMS therapy.

**Table 1:** Demographic, clinical, and treatment characteristics (*n* = 27).

	**Site 1: Butler (** ***n*** **=** **16)**		**Site 2: Salience (** ***n*** **=** **11)**	
	**Range or number**	**Mean (SD) or %**	**Range or number**	**Mean (SD) or %**
Depression severity (IDS-SR total)	34–71	47	25–61	37
Depression severity (PHQ-9)	9–27	19	7–27	15
Age (years)	22–78	48	25–71	49
Sex (Female)	13	81%	6	56%
Past (Lifetime) ECT treatment	3	19%	0	0%
Past (Lifetime) psychiatric hospitalization	11	69%	2	20%
TMS therapy in past (Lifetime) depressive episodes	4	25%	5	46%
Level of prior meditation experience (0–10 scale)	0–9	3.2	0–8	4.3
Total number of MCBT+TMS sessions	1–25	17	6–25	20
Number of TMS sessions in this course	14–48	36	14–36	34

As shown in Table 2, a comparison between baseline and endpoint scores confirmed a significant mean reduction in depression symptoms from baseline to the last observation carried forward (LOCF) on the IDS-SR and PHQ-9 scales, as expected in open-label treatment. At the end of their treatment courses, perceived stress was significantly diminished (*p* < 0.001), and quality of life (*p* < 0.000005) and multiple aspects of mindfulness (as shown in Table 2) were significantly enhanced. Comparison of treatment outcomes between the 17 completers vs. 10 who terminated early from the study revealed equivalent outcomes between the groups (PHQ-9 % change completers vs. non-completers: 71.1 ± 14.7 % vs. 47.9 ± 42.5 %, *p* = 0.125, corrected for non-equal variances). Additionally, although shorter meditation tracks were used during weeks 3–5 at one of the sites (to accommodate TMS protocols of shorter duration), treatment outcomes were not significantly different between sites.

**Table 2:** Change in clinical measures (*n* = 17)^a^.

**Measure**	**Baseline**	**Endpoint**	***P***
Depression severity (IDS-SR)	42.42 ± 11.76	17.42 ± 11.46	<0.000001
Depression severity (PHQ-9)	17.19 ± 5.84	5.93 ± 5.22	<0.000001
Quality of life/enjoyment	37.87 ± 8.31	46.65 ± 7.87	<0.001
Perceived stress	39.78 ± 7.03	28.61 ± 8.23	<0.000005
Mindfulness—observing	24.62 ± 6.6	26.90 ± 4.68	0.021
Mindfulness—describing	24.36 ± 6.6	27 ± 6.4	0.012
Mindfulness—awareness	20.00 ± 6.7	23.14 ± 4.5	0.033
Mindfulness—nonjudgmental Inner Experience	19.14 ± 5.9	24.77 ± 6.7	0.000066
Mindfulness—nonreactivity	16.50 ± 4.4	20.05 ± 4.4	0.003
MAIA attention regulation	1.73 ± 0.83	2.45 ±−0.85	0.0007 (corrected)
MAIA self-regulation	1.70 ± 1.0	2.50 ± 1.1	0.002 (corrected)
MAIA body listening	1.36 ± 0.97	2.23 ± 1.0	0.0026 (corrected)

a *Baseline data recorded prior to beginning the first TMS session. Endpoint data recorded as individual last observation carried forward (LOCF)*.

### Feasibility

Overall, we found MBCT+TMS to be less acceptable than anticipated. Only 17 patients (59%) completed the study in the intent-to-treat sample (*n* = 27). Ten subjects withdrew early for the following reported reasons: increased anxiety levels (*n* = 3), losing interest in meditative practices (*n* = 1), becoming angry or irritated by the audiotracks (*n* = 1), disliking the content of the tracks (*n* = 1), increased tics (*n* = 1 in a patient with comorbid Tourette's syndrome), excessive time commitment (*n* = 1), concerns about falling asleep (*n* = 1), and premature termination of the TMS course for other reasons (*n* = 1). The completers and dropouts presented no significant differences at baseline on the above clinical and mindfulness measures. There were no differences between the two study sites with regard to completion rate (9/16 [56%] at Butler and 8/11 [76%] at Salience, *p* = 0.384).

Participants varied considerably with regard to the perceived benefit derived from participation in MBCT+TMS; the mean ± SD overall self-rated benefit was 4.6 ± 3.3 and ranged from 0 (no benefit) to 10 (significant benefit). Specific benefits endorsed by individual participants, in free form response surveys, included learning how to become calm (*n* = 8), attending more to thoughts and physical sensations (*n* = 3), recognizing distressing thoughts (*n* = 1), creating a power thought (*n* = 1), redirection of focus away from the sensory experience of TMS pulses (*n* = 1), and having a goal for each TMS session (*n* = 1).

Meditation during TMS was rated as moderately difficult (mean rating: 5.4 ± 3.2) by the patients, recorded on a self-report scale ranging from 0 (“no-effort”) to 10 (“impossible”). The MBCT tracks were endorsed as efficient for gaining mastery in meditation by 65.2% of the subjects. Thirteen (58.3%) participants continued to meditate either “sometimes” (*n* = 8) or “often” (*n* = 5) during their TMS stimulation sessions after guidance from the audiotapes ended.

**Figure 2:**
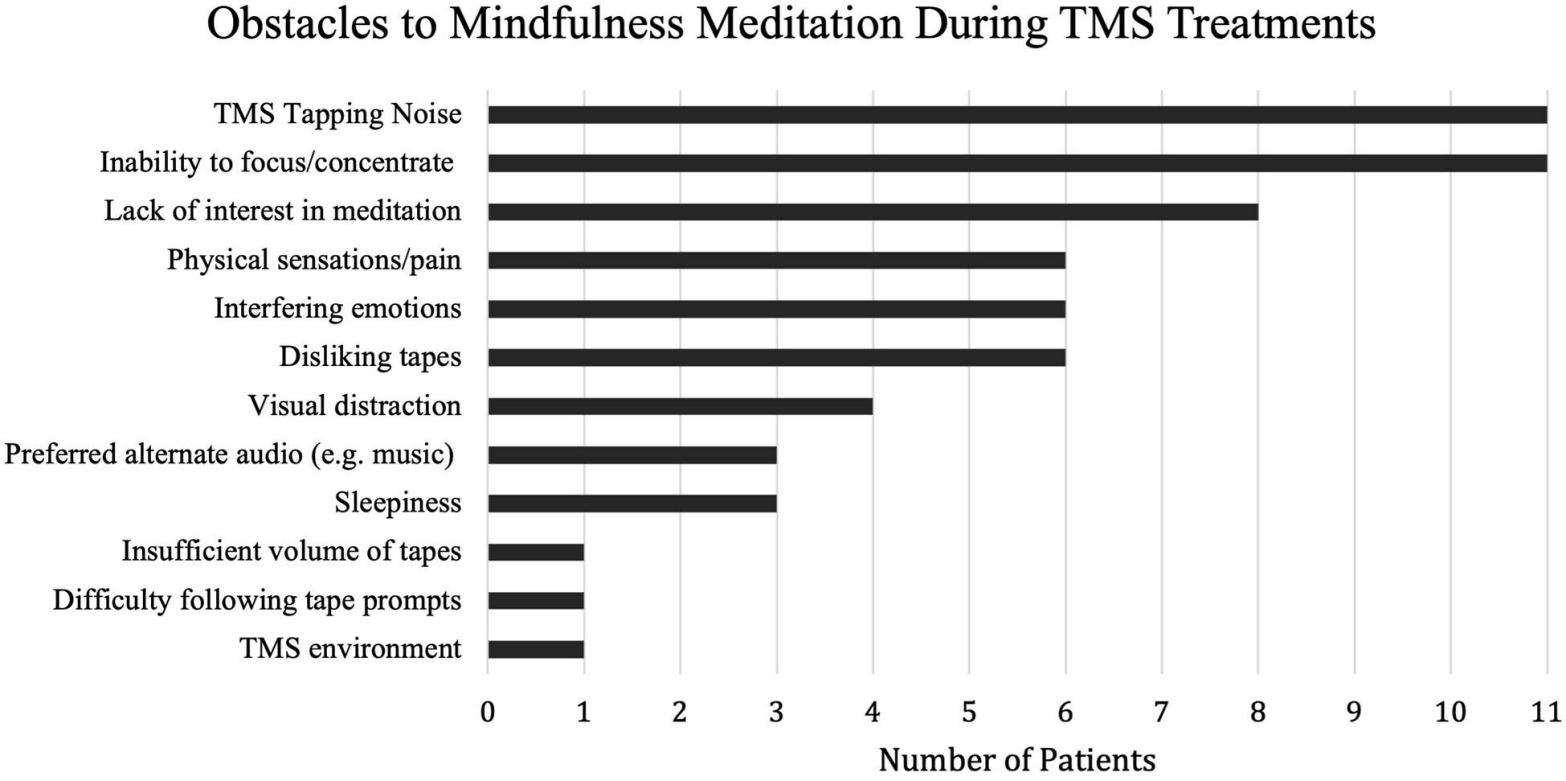
Feasibility Questionnaire Participant Responses. TMS = Transcranial Magnetic Stimulation.

Furthermore, feasibility questionnaire responses also indicated that more than a majority (71%) of participants experienced one or more days when they did not want to meditate during their TMS sessions. Reasons included (1) the induction of stressed or anxious states when meditating during TMS (*n* = 4), (2) dislike of the audiotape or a specific mindfulness exercise (*n* = 4), (3) concerns about falling asleep (*n* = 3), and (4) difficulty concentrating due to the percussive noise and sensation of TMS pulses (*n* = 2). Patients were also asked about the specific obstacles to meditation encountered during TMS sessions, summarized in Figure 2.

Only 14 (58.3%) participants reported practicing meditation at home or elsewhere outside of the TMS clinic, as encouraged by the study protocol and research staff. Similar issues were identified, i.e., difficulty focusing/concentrating (*n* = 7), time commitment (*n* = 5), lack of motivation (*n* = 3), lack of interest (*n* = 2), difficulty finding a quiet place to meditate (*n* = 2), forgetting to meditate (*n* = 2), and anxiety (*n* = 1), along with the unavailability of the meditation audiotracks outside of the TMS clinic (*n* = 1) and lack of sufficient meditation skill acquisition (*n* = 1).

Further evidence indicating poor feasibility of the concurrent combination of TMS and MBCT came from the fact that 16 subjects (69.6%) expressed their intent to continue meditating after the end of the study, yet only nine (37.5%) indicated they would want to meditate during TMS sessions again in the future if they underwent another course of TMS.

Patients provided recommendations for improving the TMS+MBCT experience: diversifying the types of meditation exercises (*n* = 4), increasing the volume of the audio tracks (*n* = 1), reformatting the audio tracks to reduce silent pauses (*n* = 2), and choosing new MBCT exercises that are less prone to let subjects fall asleep during treatment (*n* = 2). While those recommendations might be feasible, two suggestions of changing the location (*n* = 1) of meditation and adjusting the sitting posture (*n* = 1) while undergoing TMS are intractable for a future TMS/MBCT paradigm due to the patient position necessary for adequate TMS coil contact.

## Discussion

We conducted a dual-site, open-label pilot study of MBCT during TMS for depression to evaluate preliminary feasibility, acceptability, and clinical outcome of this combined paradigm. Mindfulness training has been shown to affect top-down regulation of emotional processing, with diminished activation of the DLPFC during negative emotional states (Chiesa et al., 2013). Standardized MBCT previously demonstrated the efficacy for sustaining remission from a major depressive episode (Williams et al., 2014), motivating us to consider that audio-guided meditation exercises during TMS therapy sessions delivered to DLPFC might be a feasible dual therapy for TMS patients. This notion was strengthened by reports of positive outcomes when TMS was delivered concurrently with psychotherapy for MDD (Donse et al., 2018). Wearing some form of ear protection (earplugs or earbuds) is required during TMS treatments, and most TMS patients listen to music or audiobooks on earbud headphones during their sessions. If it proved feasible and worked synergistically with TMS therapy to enhance outcomes, administering a concurrent therapeutic intervention like MBCT through narrated meditation audio files might be an efficient way to use the 20–40 min each day when patients are reclined and receiving stimulation.

We modified the necessary elements of an established MBCT program for application to patients with MDD undergoing daily TMS treatments for 5 weeks in two clinics, and we provided a general structure and support for participants to engage in MBCT during each TMS session. We found the feasibility and acceptability of the combined treatment approach to be poor, as indicated by the high number of dropouts and the feedback data we collected on feasibility questionnaires.

Despite the use of earbud headphones designed to minimize ambient sounds, clicking noise and sensory input from the TMS coil were significant barriers to engaging in the mindfulness exercises. This issue arose during stimulation with both rapid (10–20 Hz) and slower (1 and 5 Hz) pulse frequencies. The efforts to maximize the volume of the tracks, using multiple different electronic devices, did not appear to overcome this problem, which was likely related to competing for sensory inputs beyond just the sounds associated with TMS. Percussive sensations on the cranium, along with contractions of the scalp and face muscles, are significant inputs particularly among patients with high MTs who receive stimulation at relatively high intensities. These concerns appear relevant to MBCT and were not entirely predictable, given that TMS patients routinely choose to listen to music or audiobooks through earbud headphones during their treatment sessions and do so without apparent distress.

The use of an audio-recorded guide through sequential steps of each meditation exercise was often associated with increased anxiety and psychological discomfort, in some cases causing early termination. Negative feelings would usually be discussed with teachers during MBCT encounters, but in our modified version of MBCT+TMS protocol, the patients did not have a substantive relationship with an MBCT therapist. This appeared to be a shortcoming of our approach that negatively impacted the treatment outcomes, though adverse events are reported in up to 10.6% of participants even in evidence-based mindfulness-based programs (Baer et al., 2019).

While MBCT appears less than feasible during the delivery of TMS, future work can assess the appropriateness of “offline” MBCT during a course of TMS, i.e., with meditation exercises directly prior to or following the stimulation session. This approach would overcome the limitation of TMS sensory input, and it would provide an important context to the current report of anxiety and psychosocial impediments contributing to subject termination. Both online and offline concurrent cognitive therapies appear to improve TMS outcomes across a range of psychiatric disorders; it remains to be seen whether MBCT can moderate an improvement in this context as a dual therapy for depression. The potential for synergistic effects of TMS delivered sequentially (rather than simultaneous) with other therapeutic interventions is highlighted across numerous studies such as TMS paired with cognitive training for dementia (Bagattini et al., 2020) and TMS paired with physiotherapy for poststroke rehabilitation (Moslemi Haghighi et al., 2021). While a sequential delivery of TMS and MBCT may prove more feasible than the protocol we studied, the time commitment for daily TMS therapy is already substantial so the burden of extra time in the clinic for meditation exercises might deter some patients.

Future investigations evaluating a potential synergistic role for MBCT with TMS therapy should also examine the longer-term outcomes to understand potential therapeutic synergy. According to Segal et al. (2019), the investment in practice while learning the MBCT protocol is predictive of the commitment after the 8 weeks of program participation, with a steady increase of metacognitive capacities, recognition of reward and positive affect, and regulation of mood states during the following 2 years. Additionally, it remains unseen how the cognitive manipulations of MBCT may interact with the established improvements in cognitive and cortical function following the effective TMS therapy (Spampinato et al., 2013). The negative psychosocial incidents reported here may pose a hindrance to cognitive modulation in future dual-therapy paradigms.

Limitations of this study may include modest sample size and the use of self-report measures, though it is not clear whether more objective measures would meaningfully contribute to the results since feasibility and acceptability were the primary outcomes of interest. Given the open-label nature of the trial, we are unable to draw any conclusions of the impact of the combined intervention on clinical outcomes such as degree of symptom reduction, or likelihood of achieving remission. The magnitude of change in depression severity we observed following the course of TMS is similar to that we have reported for other open-label trials (Philip et al., 2016; Russo et al., 2018).

## Conclusion

This pilot study underscored feasibility issues surrounding concurrent TMS administration and mindfulness-based practice in the form of listening to audio-guided meditation exercises. It is possible that learning and practicing MBCT immediately before or immediately after TMS treatment sessions would have worked better for achieving the goals we identified; these approaches merit evaluation in future trials. Acquisition of meditation skills in a more conducive environment, at least initially, seems like an important foundation for successful use of meditation in physically uncomfortable and highly distracting situations such as one finds oneself during a TMS therapy session.

## Data Availability Statement

The raw data supporting the conclusions of this article will be made available by the authors, without undue reservation.

## Ethics Statement

The studies involving human participants were reviewed and approved by Butler Hospital Institutional Review Board. The patients/participants provided their written informed consent to participate in this study.

## Author Contributions

FC, LC, and ET conceptualized and designed the study. FC, MG, ET, and FK (Butler) and ND, DS, and JK (Salience) conducted study interventions, collected data and managed the database. FC, LC, FK and MG conducted statistical tests. FC drafted the first manuscript, and all coauthors contributed input and revisions to the manuscript. All coauthors read and approved the submitted version.

## Conflict of Interest

LC serves as scientific advisor to Neuronetics Inc., Nexstim PLC, Affect Neuro Inc., Neurolief LTD, Sage Therapeutics, Otsuka, and Janssen Pharmaceuticals Inc. She has received research support (to Butler Hospital) from Neuronetics Inc., Neosync Inc., Nexstim PLC, Affect Neuro Inc., and Janssen Pharmaceuticals Inc. The remaining authors declare that the research was conducted in the absence of any commercial or financial relationships that could be construed as a potential conflict of interest.

## Publisher's Note

All claims expressed in this article are solely those of the authors and do not necessarily represent those of their affiliated organizations, or those of the publisher, the editors and the reviewers. Any product that may be evaluated in this article, or claim that may be made by its manufacturer, is not guaranteed or endorsed by the publisher.
